# Establishment of a 3D *In Vitro* Model to Accelerate the Development of Human Therapies against Corneal Diabetes

**DOI:** 10.1371/journal.pone.0168845

**Published:** 2016-12-22

**Authors:** Shrestha Priyadarsini, Akhee Sarker-Nag, Tyler G. Rowsey, Jian-Xing Ma, Dimitrios Karamichos

**Affiliations:** 1 Department of Ophthalmology/Dean McGee Eye Institute, University of Oklahoma Health Sciences Center, Oklahoma City, Oklahoma, United States of America; 2 Department of Biology and Chemistry, East Central University, Ada, Oklahoma, United States of America; 3 Department of Physiology Harold Hamm Diabetes Center, University of Oklahoma Health Sciences Center, Oklahoma City, Oklahoma, United States of America; Cedars-Sinai Medical Center, UNITED STATES

## Abstract

**Purpose:**

To establish an *in vitro* model that would mirror the *in vivo* corneal stromal environment in diabetes (DM) patients.

**Methods:**

Human corneal fibroblasts from Healthy (HCFs), Type 1DM (T1DM) and Type 2DM (T2DM) donors were isolated and cultured for 4 weeks with Vitamin C stimulation in order to allow for extracellular matrix (ECM) secretion and assembly.

**Results:**

Our data indicated altered cellular morphology, increased cellular migration, increased ECM assembly, and severe mitochondrial damage in both T1DM and T2DMs when compared to HCFs. Furthermore, we found significant downregulation of Collagen I and Collagen V expression in both T1DM and T2DMs. Furthermore, a significant up regulation of fibrotic markers was seen, including α-smooth muscle actin in T2DM and Collagen III in both T1DM and T2DMs. Metabolic analysis suggested impaired Glycolysis and Tricarboxylic acid cycle (TCA) pathway.

**Conclusion:**

DM has significant effects on physiological and clinical aspects of the human cornea. The benefits in developing and fully characterizing our 3D *in vitro* model are enormous and might provide clues for the development of novel therapeutics.

## Introduction

Diabetes mellitus (DM) is a common metabolic disease characterized by hyperglycemic condition that has a higher prevalence rate with increased number of cases every year[1,2]. Approximately 371 million people have been diagnosed with DM worldwide and the incidence rate is expected to double by 2030[3–5]. In the United States it has also been termed as the epidemic disease of an increasing age and obese population[1]. Approximately 6.2 million people are underdiagnosed in the United States alone.

DM is broadly divided into two main categories: Type 1DM (T1DM) and Type 2DM (T2DM). T1DM is known as “insulin dependent” or “juvenile-onset’ diabetes and caused due to the autoimmune destruction of the β-cells in the pancreas, accounting for about 5–10% of total DM cases worldwide[2,5,6]. T2DM, on the other hand, is known as “non-insulin dependent” or “adult-onset” diabetes, caused by excessive elevated blood glucose levels that lead to insulin resistance. T2DM accounts for about ~90–95% of total DM population [2,5,6].

Chronic hyperglycemic conditions during DM often lead to complications, damage, and failure of several different organs including the eyes, heart, nerves, kidney and blood vessel. The most common ocular complications during DM include diabetic retinopathy, cataract, glaucoma, ischemic optic neuropathy, cranial nerve palsies and recurrent corneal erosion syndrome [7–11]. The cornea, in particular, is greatly affected with changes and defects that include recurrent corneal erosions, persistent epithelial defects, corneal endothelial damage, reduced corneal sensitivity, increased corneal thickness, susceptibility to corneal trauma and alteration in tear quality and quantity [7–9]. To date, studies on DM-related corneal defects, commonly known as diabetic keratopathy, have been primarily focused on the epithelial layer and nerves that are known for significant damages and deterioration [7–9,12,13]. These studies are mainly *in vivo* with the exception of Dr. Ljubimov’s and co-authors model where cadaveric corneas are used to study epithelial defects [13,14]. While these studies have significantly increased our knowledge with regards to the pathophysiology of diabetic keratopathy, we are still lacking a good grasp of understanding the molecular mechanism involved. As a result, any developed therapeutic agents and protocols that have worked in rodents have failed in humans [15–17].

We have developed a stroma-like model that consists of primary human corneal fibroblasts from healthy (HCF), T1DM, and T2DM donors that can mimic the stroma seen *in vivo*. In this study, we investigated the morphological and molecular differences between healthy and diabetic self-assembled extracellular matrix (ECM). To the author’s knowledge, this is the first *in vitro* model available which can be used to recapitulate the *in vivo* corneal stromal defects resulted by diabetic keratopathy. Further studies of such a novel model may enable development of novel therapeutics to treat corneal DM.

## Materials and Methods

### Ethics and inclusion criteria

Institutional review board approval was received prior to initiation of experiments described in this study (#4509). All parts of the study met the tenets of the Declaration of Helsinki. Corneal samples were obtained from the National Development and Research Institute (NDRI) and the Oklahoma Lions Eye Bank. Inclusion criteria for the diabetic donors included clinical diagnosis of Type 1 or 2 diabetes and absence of other unrelated diseases or ocular pathology. The control group included corneas isolated from cadavers with no history of ocular trauma or systemic diseases (Table 1). The causes of death for the diabetic groups were considered to be diabetic-related complications (acute cerebral infarction, cerebrovascular accident, complications from end stage renal disease, respiratory failure) with the cause of death for healthy controls varying from accidental to non-diabetic related diseases (Blunt force trauma, head trauma, end stage renal disease, acute segment elevation myocardial infarction, subarachnoid hemorrhage, cardiac arrest). In this study a total of 16 diabetic donors’ corneal samples (8 donors for each T1DM and T2DM with a total of 4 males and 4 females in each group) and 8 healthy control samples (5 males and 3 females) were analyzed. The average age range for each group was as follows: (Healthy) 57.7±5.8 years, (T1DM) 55±6.8 years, and (T2DM) 59.14±4.94 years. The duration of diabetes was from 3–30 years (with a mean of 15.71±4.17 years) (Table 1).

**Table 1:** Characteristics of study population

	Healthy Controls	T1DM	T2DM
**Donors (n)**	**8**	**8**	**8**
**Range(years)/Mean Age**	**30-76/57.71429**	**23-70/55**	**40-73/59.14286**
**Sex (male/female) (n)**	**5/3**	**4/4**	**4/4**
**Diabetics duration range**	**-**	**3–28**	**4–30**

### Primary culture of healthy and diabetic corneal fibroblast cells

HCF, T1DM and T2DM cells were isolated and cultured in Eagle’s Minimum Essential Medium (American Type Culture Collection, Manassas, VA, USA) containing 10% fetal bovine serum (Atlantic Biologicals, Miami, FL, USA) and 1% antibiotic (Life Technologies, Grand Island, NY, USA) [18–21]. The cultures were passed upon 80–100% confluence following previously optimized protocols [18–21].

### Assembly of 3D constructs

3D constructs for HCFs, T1DM, and T2DM cells were prepared as previously described [18,19,21,22]. Briefly, 1 × 10^6^ cells/well were seeded on polycarbonate membrane inserts with 0.4-μm pores (Corning Costar; Corning Incorporated, Corning, NY, USA) and cultured in Eagle’s Minimum Essential Medium containing 10%fetal bovine serum and 1% antibiotic and stimulated with 0.5 mM 2-O-α-Dglucopyranosyl-L-ascorbic acid (American Custom Chemicals Corporation, San Diego, CA, USA). Cultures were maintained for 4 weeks and fresh media was supplied every other day during the entire study period.

### Real-time PCR

mRNA expression of our samples was evaluated by qRT-PCR as previously described [20,22,23]. Total RNA was extracted using Ambion RNA mini extraction kit (Ambion TRIzol Plus RNA Purification Kit: Life technologies, Carlsbad, CA) followed by cDNA synthesis using SuperScript III First-Strand Synthesis SuperMix (Invitrogen, Carlsbad, CA) as per manufacturer’s protocol. TaqMan gene expression of (Applied Biosystems, Foster City) GAPDH (Hs99999905_m1) and 18S (Hs99999901_s1) was used as our controls. ACTA2 (Hs00426835_m1), COL1A1 (Hs00164004_m1), COL3A1 (Hs00943809_m1), COL5A1 (Hs00609133_m1), IGF1 (Hs01547654_m1) and IGF1R (Hs00609566_m1) were investigated (Table 2). Data analysis was performed using Graph Pad Prism 6 and MS-Excel.

**Table 2:** RT-PCR probes and their dilutions

Probes	Catalogue No#1	Final concentration	Company
**GAPDH**	**Hs99999905_m1**	**1X**	**Life technologies**
**18S**	**Hs99999901_s1**	**1X**	**Life technologies**
**ACTA2**	**Hs00426835_m1**	**1X**	**Life technologies**
**COL1A1**	**Hs00164004_m1**	**1X**	**Life technologies**
**COL3A1**	**Hs00943809_m1**	**1X**	**Life technologies**
**COL5A1**	**Hs00609133_m1**	**1X**	**Life technologies**
**IGF1**	**Hs01547654_m1**	**1X**	**Life technologies**
**IGF1R**	**Hs00609566_m1**	**1X**	**Life technologies**

### Western blot analysis

Cell lysates were used for western blot analysis, as per our previously optimized protocol [20,24]. Preparation of cell lysates was initiated by using RIPA buffer (50 mM Tris, pH 8, 150 mM NaCl, 1% Triton X-100, 0.1% SDS, 1% sodium deoxycholate) containing protease and phosphatase inhibitors (Sigma Aldrich; St. Louis, MO) followed by brief incubation, centrifugation and stored at -20°C until further usage. Equal amounts of proteins were loaded on to 4%–20% Tris-Glycine gels (Novex, Life technologies, Carlsbad, CA) for gel electrophoresis followed by protein transfer to a nitrocellulose membrane (Novex, Life Technologies) and further incubation in 5% BSA blocking solution (Thermo Scientific). The membranes were then incubated with primary antibodies (Table 3): anti-Collagen I (ab34710; Abcam; Cambridge; MA), Collagen III (ab7778; Abcam; Cambridge; MA), Collagen V (ab94673; Abcam; Cambridge; MA), α-smooth muscle actin (ab5694; Abcam; Cambridge; MA), IGF1(Cambridge; MA), 1GF1R (Abcam; Cambridge; MA), glyceraldehyde 3-phosphate dehydrogenase (GAPDH, ab9485; Abcam; Cambridge; MA) (Table 3). Antibodies were used at 1:1000 dilution in TBST overnight at 4°C with rocking followed by washing of the membranes and incubation with a secondary antibody (Alexa Flour® 568 Donkey anti-Rabbit, IgG [H+L], Abcam) at 1:2000 dilutions for 1 hr. UVP imaging system was used for band detection and quantification by densitometry. Net intensities were normalized to the loading control (GAPDH) and depicted as fold change.

**Table 3:** Western Blot antibodies and their dilutions

Antibody	Catalogue No#	Dilution	Company
**Anti-Collagen I**	**ab34710**	**1/1000**	**Abcam**
**Anti-Collagen III**	**ab7778**	**1/1000**	**Abcam**
**Anti-Collagen V**	**ab94673**	**1/1000**	**Abcam**
**Alpha-smooth muscle actin**	**ab5694**	**1/1000**	**Abcam**
**Insulin Growth Factor 1(IGF1)**	**ab9572**	**1/1000**	**Abcam**
**Insulin Growth Factor Receptor 1**	**ab131476**	**1/1000**	**Abcam**
**Anti-GAPDH**	**ab9485**	**1/1000**	**Abcam**

### Immunofluorescence

The constructs were processed for cytochemical analysis by fixing with 4% paraformaldehyde in PBS [18,22,25,26]. This was followed by sample permeabilization with PBS containing 0.25% Triton X-100 (Sigma-Aldrich, St. Louis, MO, USA) and blocking with 1% BSA (Thermo Scientific) in PBST (0.05% Tween 20 (Sigma-Aldrich, St. Louis, MO, USA) PBS) containing 0.3 M glycine (Fischer Scientific,). Samples were incubated with Alexa Flour Phalloidin (Life Technologies, USA) primary antibody overnight at 4°C and stained with DAPI (1μg/ml, Life Technologies, USA). The samples were imaged using confocal microscope (Olympus Confocal FV 500, USA).

### Cell migration and proliferation assay

Cell migration was assessed using the *in vitro* scratch assay model [27]. Briefly, 1×10^6^ cells/well for all three cell types (HCFs, T1DM and T2DM) were seeded in six well plates and allowed to attain 100% confluency. Scratches were performed using a sterile micropipette tip through the cell monolayer and washed with PBS after the scratch was made. This was followed by adding fresh medium to the culture plates and then imaging the wounded site at predetermined time points (0hr, 4hr, 24hr and 48hr). The migration pattern was evaluated and quantified using ImageJ software.

Cellular proliferation was measured using vybrant MTT cell proliferation assay kit (Life Technologies, USA). About 1× 10^5^ cells/well was seeded and after 24 hours of culture 10μl of the 12mM MTT stock solution was added to each well and incubated for another 3–4 hours at 37°C as per the manufacturer’s protocol. Thereafter, 100μl of the SDS-HCl solution was added to each well and evenly mixed, followed by a brief incubation for about 4 hours at 37°C in a humidified chamber. Samples were mixed again and the absorbance was measured at 570nM using the plate reader. The results were analyzed and processed in Excel and Graph Pad Prism 6 (GraphPad Software, CA) to determine the cellular proliferation rate.

### Transmission Electron microscopy and mitochondrial quantification

All 3D constructs were fixed with 4% Paraformaldehyde and 2% Glutaraldehyde in 0.1M Sodium Cacodylate buffer for 6 days at 4°C. Samples were further post fixed in 1% Osmium tetroxide in Sodium Cacodylate, and rinsed with 0.1M Sodium Cacodylate buffer. Following previously reported methods [18,22,28,29] samples were embedded in resin plus BDMA (accelerator) and polymerized at 60°C for 48 hours. Ultrathin sections were stained with Lead Citrate and Uranyl Acetate before viewing on a Hitachi H7600 Transmission Electron Microscope at 80 kV equipped with a 2k x 2k AMT digital camera. Mitochondrial structure was imaged and quantified. Normal mitochondrial structure was defined as clear double membrane, discrete cristae and little internal space. The number of mitochondria that did not exhibit the normal mitochondrial structure were plotted and quantified.

### Metabolomics

The samples were prepared as per previously optimized protocol [30–32]. Briefly, samples were washed with 1xPBS, lysed with ice-cold 80% methanol, incubated briefly on dry ice for about 15 minutes and then homogenized for ensuring complete cell lysis. Isolated metabolites were stored at -80°C until further processing. CIMminer was used to analyze the metabolomics data which was available from the genomics and bioinformatics group (http://discover.nci.nih.gov/cimminer/home.do) for generating a color-coded Clustered Image Map (CIM). For all the metabolites, the raw net intensity data present in at least n = 3 were input into the freely available software for generating a one matrix CIM. All the metabolites were clustered according to abundance.

### Statistical Analysis

Statistical analysis was conducted using Graph Pad Prism 6 and a one-way ANOVA and Mann-Whitney unpaired T-test, where appropriate. P<0.05 was considered statistically significant.

## Results

### ECM and cellular morphology in T1DM and T2DM

**Fig 1:**
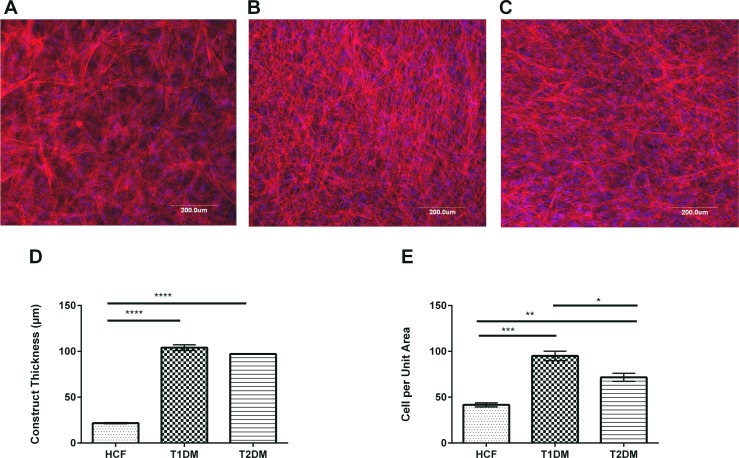
Confocal microscopy showing cellular morphology of HCFs, T1DMs, and T2DMs when cultured in our 3D constructs. (A-C) Representative images of HCFs, T1DMs, and T2DMs stained with Phalloidin (red) and DAPI (blue). (A) HCF cells morphology showed uniform fibril diameter with regular spacing. (B) T1DM cells showed thin fibril morphology with no interfibrillar spacing. (C) T2DM cells morphology also showed lack of fibrils organization and arrangement. (D) Quantification of the construct thickness in HCFs, T1DMs, and T2DMs. (E) Cells per unit area (mm^2^) in HCFs, T1DMs, and T2DMs. For all the three cell types a minimum of 4 confocal z-stack images were used, which were averaged, plotted and analyzed by Graph Pad Prism 6 software. Error bars represent standard error of the mean. One-way ANOVA was performed (* = p≤0.05; ** = p≤0.01; *** = p≤0.001; **** = p≤0.0001).

We have previously reported that HCFs cultured in Vitamin C on transwell polycarbonate membranes lead to multi-layer, highly organized 3D construct [18,19,21–23,29,33]. In this current study, we extended our expertise to develop those 3D constructs using T1DM and T2DM primary human corneal cells. Fig 1A–1C shows representative images of cells stained with Phalloidin (red) and DAPI (blue).

Both our T1DM and T2DM constructs (Fig 1) showed significant increase in thickness (4.5 fold; p≤0.0001 and 4 fold; p≤0.0001 respectively) when compared to HCFs (Fig 1D). In addition, there was a significant increase in cells per unit area (mm^2^) in both T1DM and T2DM (Fig 1E) when compared to HCFs, indicating higher proliferation rates.

### Cellular migration and proliferation

**Fig 2:**
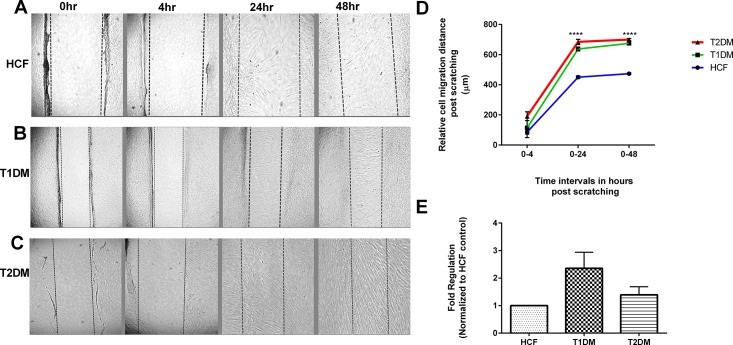
Scratch wound healing and MTT assay. (A-C) HCF, T1DM and T2DM cells were scratched and the relative cell migration distance was quantified at 0hr, 4hr, 24hr and 48hr time points. (D) Scratch assay quantification showing significant increase in cell migration distance in both T1DM and T2DMs when compared to HCFs. (E) MTT assay quantification for HCF, T1DM, and T2DMs. Data was normalized to HCFs and fold regulation is plotted. One way ANOVA for total n≥3 data sets. (* = p≤0.05; ** = p≤0.01; *** = p≤0.001; **** = p≤0.0001).

Studies have already shown hyperglycemic conditions often accelerates cellular proliferation [34–37]. We analyzed and quantified cell migratory pattern for all the three cell types following the observation of increased ECM assembly (Fig 1). Using *in vitro* scratch assay model (Fig 2) we observed significant increased cellular migration of 4 fold (p≤0.0001) in both T2DM (Fig 2C and 2D) and T1DM constructs (Fig 2B and 2D) when compared to HCFs (Fig 2A and 2D). These data suggest that the ECM secreted by DM cells is altered in a way that promotes hyper cellular migration.

Furthermore, we tested cellular proliferation using MTT assay, as shown in Fig 2E. T1DMsshowed almost a 3 and 2 fold upregulation when compared to HCFs and T2DMs respectively.These findings did not reach significance, but suggest a trend for increased cell proliferation.

### mRNA expression of secreted collagens by T1DM, T2DM and HCFs

**Fig 3:**
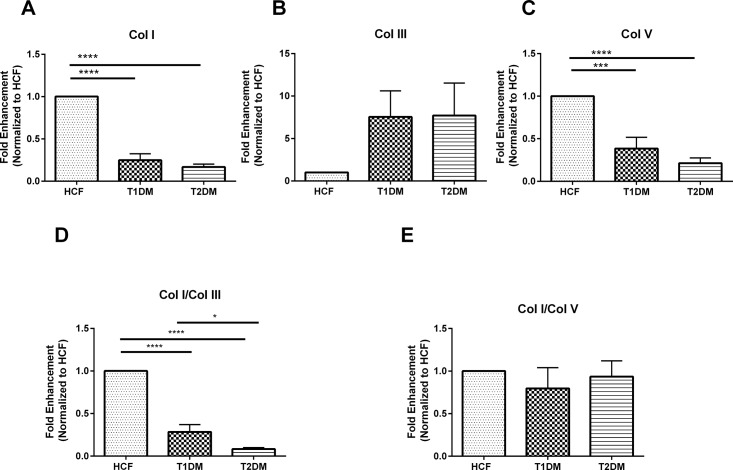
**mRNA expression for (A) Col I, B) Col III, C) Col V, D) Col I/Col III ratio, and E) Col/Col V ratio, in HCFs, T1DMs, T2DMs**.(A-E) Quantification of gene expression and their ratios that are normalized to the loading control. n≥7 for HCFs, T1DMs and T2DMs. Error bars represent standard error of the mean. One-way ANOVA was performed (* = p≤0.05; ** = p≤0.01; *** = p≤0.001; **** = p≤0.0001).

We performed qRT-PCR to investigate Col I, Col III, and Col V expression by HCFs, T1DMs, and T2DMs. Significant downregulation by 4 fold (p≤0.0001) in Col I expression was seen in both T1DM and T2DMs when compared to HCFs (Fig 3A). Similarly, in T1DMs Col V expression was decreased by 3 fold (p≤0.001) and by 4 fold (p≤0.0001) in T2DMs (Fig 3C). Surprisingly, Col III regulation did not reach significance; although the trend was similar to both T1DM and T2DMs expressing higher levels of Col III by at least 2 fold (Fig 3B).

The data was also plotted and examined as Col I/Col III and Col I/Col V ratios. Col I/Col III ratio was 4 fold (p≤0.0001) downregulated in T1DMs and 4.5 fold (p≤0.0001) downregulation in T2DMs. No significant difference was observed in Col I/Col V ratio in any of the cell types/constructs. Overall, this data indicated that the constructs with T1DM and T2DM cells were progressively becoming more fibrotic.

### Protein level Collagen expression by T1DM, T2DM and HCFs

Protein levels were also examined for all three cell types. While complete agreement with mRNA data is ideal, differences are not unusual, mainly attributed to the temporal effects involved in mRNA expression that may not reflect protein expression at the functional level [38]. In post-transcriptional modification where correlation between mRNA and protein expression primarily depends upon the type of cell and the gene that is regulated [39].

**Fig 4:**
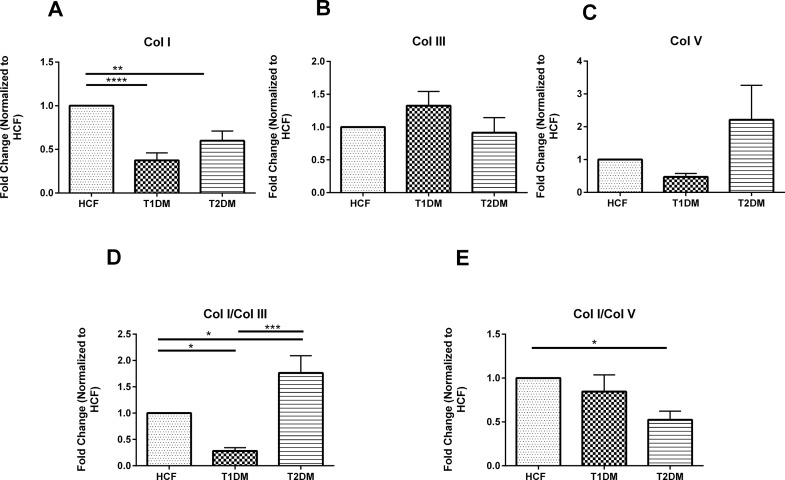
**Protein expression for (A) Col I, B) Col III, C) Col V, D) Col I/Col III ratio, and E) Col/Col V ratio, in HCFs, T1DMs, T2DMs**.(A-E) Quantification of protein bands and their ratios that are normalized to the loading control. And, n≥7 for HCFs, T1DMs and T2DMs. Error bars represent standard error of the mean. One-way ANOVA was performed (* = p≤0.05; ** = p≤0.01; *** = p≤0.001; **** = p≤0.0001).

Expression of Col I was significantly downregulated in both T1DM and T2DM cells, when compared to HCFs, by 2.5 fold (p≤0.01) in T1DMs and 1.5 fold (p≤0.05) in T2DMs. No significant difference in Col V expression was observed in HCF, T1DM or T2DMs (Fig 4C). Furthermore, we did not observe any significant differences in Col III expression. When collagen ratios were examined, T1DM showed significant downregulation (p≤0.05) in Col I/Col III ratio (Fig 4D) whereas T2DM cells showed significant upregulation (p≤0.05) when compared to the HCFs. There was also significant difference (~3 fold; p≤0.001) between the T1DM and T2DM cells Col I/Col III ratio.

### α- SMA, IGF1 and IGF1R expression by T1DM, T2DM and HCFs

**Fig 5:**
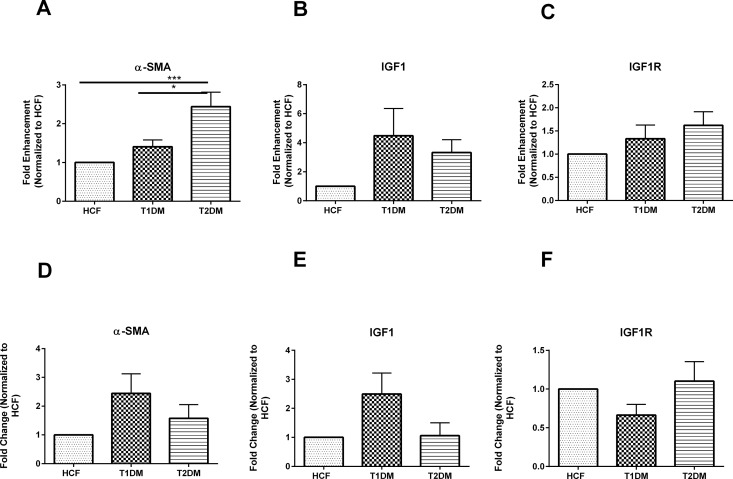
**mRNA expression for A) α-SMA, B) IGF1 and C) IGF1R in HCFs, T1DMs, and T2DMs. Protein expression for D) α-SMA, E) IGF1 and F) IGF1R in HCFs, T1DMs, and T2DMs**. (A-C)Quantification of gene expression and (D-E) quantification of protein bands normalized to HCFs where n≥7 for HCFs, T1DMs and T2DMs. Error bars represent standard error of the mean. One-way ANOVA was performed (* = p≤0.05; ** = p≤0.01; *** = p≤0.001; **** = p≤0.0001).

We further assessed cellular differences by examining the expression of the fibrotic marker α- SMA and key mediators in DM Insulin growth factor-1 (IGF1) and its receptor (IGF1-R). Our data showed significant upregulation (p≤0.01) in α- SMA gene expression in T2DMs when compared to the HCFs (Fig 5A). α- SMA expression between T1DM and T2DM constructs was also found to be significantly regulated by 1.5 fold (p≤0.05; Fig 5A). No significant difference was observed in α- SMA at the protein level (Fig 5D). Despite the role of IGF-1 and IGF-1R in DM we did not observe any significant changes either one of them at mRNA (Fig 5B and 5C) or protein levels (Fig 5E and 5F). These results suggest that IGF1and IGF1R activity might be transient in the human diabetic keratopathy.

### Altered metabolic profile of T1DM and T2DM cells

**Fig 6:**
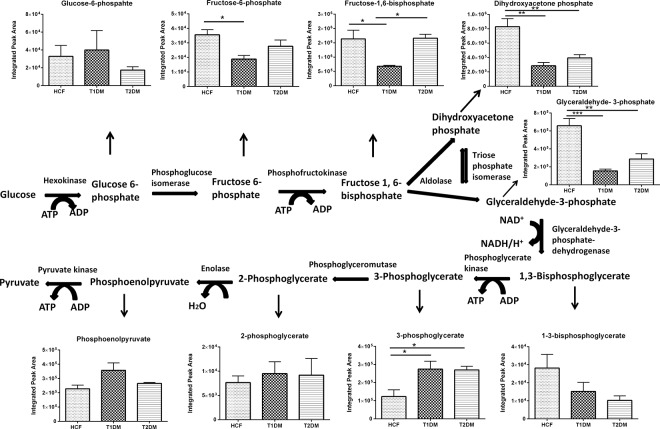
Schematic representation and quantification of the metabolic flux modulation in glycolysis cycle for HCFs, T1DMs, and T2DMs, when cultured in our 3D model. Quantification of metabolites activity expressed along the glycolysis cycle. One way ANOVA was performed for total n≥4 data sets (* = p≤0.05; ** = p≤0.01; *** = p≤0.001; **** = p≤0.0001).

The metabolic profiles of all 3D constructs were examined. Our study showed differential regulation for most of the key metabolites along the glycolytic pathway (Fig 6). Specifically, we observed changes in metabolites expression from the very first step of glycolysis where glucose gets converted to glucose-6-phosphate in both T1DM and T2DMs when compared to HCFs (Fig 6). Fructose-6-phosphate is the second step of the pathway and it was found to be significantly downregulated in T1DMs (p≤0.05) (Fig 6) when compared to HCFs. Fructose-6-phospahte is formed due to rearrangement of Glucose 6-phosphate and this step is a reversible process during normal condition of a cell. Fructose-1,6-bisphosphate was also downregulated in T1DMs (p≤0.05) when compared to both HCF and T2DMs. Significant downregulated expression of Dihydroxyacetone phosphate (2.5 folds p≤ 0.01) was witnessed in both T1DM and T2DMs when compared to our controls. Furthermore, Glyceraldehyde 3-phosphate showed significant downregulation by 3.5 fold (p≤0.001) in T1DMs and by 2 folds (p≤0.01) in T2DMs when compared to our healthy controls (Fig 6). 1, 3 Bisphosphoglycerate also showed downregulated expression in both T1DM and T2DM cells whereas 3-Phosphoglycerate was seen significantly upregulated (p≤0.05) in both the diabetic cell types (Fig 6). Clearly, tight regulation of the glycolysis pathway is essential for normal cellular metabolism. Our data suggests that both T1DM and T2DMs are metabolic dysregulated.

**Fig 7:**
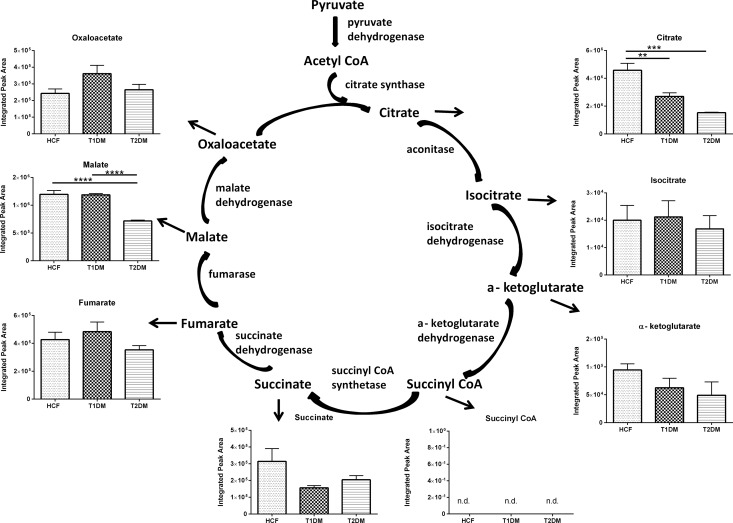
Schematic representation and quantification of the metabolic flux modulation in TCA cycle for HCFs, T1DMs, and T2DMs, when cultured in our 3D model. Quantification of metabolites activity expressed along the TCA cycle One way ANOVA was performed for total n≥4 data sets (* = p≤0.05; ** = p≤0.01; *** = p≤0.001; **** = p≤0.0001).

TCA cycle is another essential metabolic pathway which takes place inside the mitochondria of the cells and provides energy for normal functioning of the body [40–43]. Acetyl-CoA serves as the initial substrate for the TCA cycle, end products of glycolysis and pyruvate undergoes conversion and leads to the formation of acetyl-CoA. Citrate and succinate are among the vital metabolites along the TCA cycle. Citrate was significantly downregulated in T1DM (2 folds p≤0.01) and T2DM (3 folds p≤0.001; Fig 7) whereas no difference was observed in iso-citrate. Malate was also significantly downregulated in T2DM cells (3.5 folds p≤0.0001) when compared to both HCFs and T1DM cells (Fig 7). Overall, like the glycolysis pathway, these results suggest significant dysregulation of the TCA cycle in both T1DM and T2DMs.

### Structural mitochondrial damage

**Fig 8:**
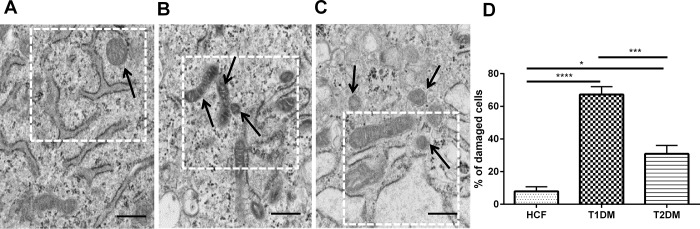
TEM image showing mitochondrial structure in HCFs, T1DMs, and T2DMs, when cultured in our 3D model. (A) HCFs: Mitochondria appeared normal, with a double membrane, discrete cristae and little internal space. (B) T1DMs: Mitochondria appeared condensed and showed expanded internal spaces. (C) T2DMs: Smaller mitochondrial structure, dilated vacuoles and presence of endoplasmic reticulum were witnessed. (D) Quantification analysis of abnormal mitochondria cells percentage in HCF, T1DM, and T2DM constructs. The data is representative of 4 independent experiments, n = 4 (* = p≤0.05; ** = p≤0.01; *** = p≤0.001; **** = p≤0.0001). Scale bars = 500nm.

Given the metabolic dysfunctions observed, we examined the mitochondrion structure while cultured in the 3D ECMs (Fig 8). We observed significant changes in mitochondrion morphology in all three cell types. Smaller mitochondrial structures in both T2DM (Fig 8C) as well as in T1DM (Fig 8B) were observed when compared to our healthy HCFs (Fig 8A). In T2DMs, the mitochondrial structures are usually smaller, which has been reported through various studies in skeletal muscle cells [40,41,44,45]. T1DMs (Fig 8B) had severe cellular mitochondrial damage with approximately 70% of the cells exhibiting some degree of abnormal structure and morphology (p≤ 0.0001; Fig 8D). T2DM (Fig 8C) cells also showed mitochondrial damage, to a lesser extent, with 30% of the cells damaged (p≤ 0.05; Fig 8D).

## Discussion

Diabetic keratopathy is one of the most common ophthalmic complications witnessed in a vast majority of DM patients [8,9,46,47] and several other corneal dysfunctions. This is mainly due to the fact that DM involves increases in corneal thickness [48–50], reduced corneal sensitivity, corneal epithelial lesions[9,46], delayed wound healing capacity and repair mechanism[51–54], and weakening of the epithelial barrier[55] leading to corneal infections and stromal fibrosis[56,57]. Other complications that have been known to be associated with DM includes but not limited to corneal nerve damage and endothelial dysfunction [58]. A diabetic patient’s prolonged exposure to hyperglycemic environment often leads to toxic end product depositions at the basement membrane which results in cell death, opacity and eventually leads to vision impairment [7,8,55,59,60] and various other ophthalmic complications.

The common treatment for treating corneal diabetes often involves corneal transplantation, corneal surgery, and inhibitor treatment and in some cases contact lenses are also prescribed[37]. However, these treatments are often not effective, mainly due to delayed wound healing in diabetics which can adversely affect the repair mechanisms of the cornea [1,7,49]. Several studies have been focused on developing various animal models that can help better understanding of the disease and also find proper treatment for DM, but treatments on these animal models have not proven to be very effective, and their value to humans has not been verified. Therefore, better understanding of corneal DM is necessary in order to discover and develop novel therapeutic targets.

In our current study, we attempted to stimulate corneal stromal cells derived from donors with DM to secrete and deposit their own organized ECM in a 3D culture model. *In vivo*, the stroma makes up approximately 90% of the corneal thickness and is composed of ECM and cells [21,33,61–63]. The ECM is made of exquisitely aligned and organized collagen, such as types I and V [18,21,61,64]. These collagens, along with various proteoglycans, are critical for the integrity and tensile strength of the stroma. Both Col I and V were present in our cultures in HCF as well as T1DM and T2DM constructs, indicating their ability to self-assemble an ECM with components similar to that found in the human stroma. Interestingly, both Col I and V mRNA levels were downregulated in T1DM and T2DM, as compared to HCFs indicating some level of dysfunction. Col III protein levels, which is associated with corneal fibrosis [18,19,21,23,33,61,64,65], was instead upregulated by T1DM and T2DM pointing towards a more fibrotic ECM been assembled. The correlation between mRNA and protein in human cells is known to be notoriously poor and it depends on various biological factors. It is unclear at this stage which is a more important finding between mRNA and protein expression for the human DM cornea. Future generation studies will aim to address this important question.

Furthermore, we determined the cell migratory properties of these cells, which is one of the crucial factors that determines effective wound healing and normal function of the cornea. It is usually directed by the interplay between various signal transduction pathways involving lipid second messengers, small GTPases, kinases, cytoskeleton-modifying proteins, and motor proteins[66]. In general, the fibroblast which essentially plays a vital role during wound healing both in *vivo* and in *vitro* often migrates to the wounding site and thereby initiates secretion of ECM proteins and effective cellular proliferation. Studies have shown that fibroblasts tend to move at a slower pace and thereby often changes their migratory direction[67]. In cornea, cellular migration which is a complex process and any deficiencies along this process can lead to either impairment and various abnormal states such as chronic inflammation, developmental defects, cancer invasion and metastasis[66]. In this study, we found significantly higher cell migration in both T1DM and T2DMs when compared to the HCFs. In agreement with what is seen *in vivo*, this suggests altered cellular processes that often lead to the secretion of aberrant ECM components.

We have previously identified several key metabolites that play a significant role during the initiation of corneal stromal defects and which clearly distinguish the differences in corneal cells derived from healthy individuals to that of the diseased ones. Not surprisingly, several metabolites were differentially regulated in both T1DM and T2DM constructs when compared to HCFs. It is already known that DM, [68,69] is associated with glucose metabolism dysfunction. Glycolysis and TCA cycle are the two most important cellular metabolic pathways that are used by the cells to generate energy for driving various bio-chemical reactions [31]. Alterations in these metabolic cycles can be critical in diabetic keratopathy. Our data shows that several key metabolites of both pathways were significantly altered which highlights the importance of our 3D *in vitro* model and validates its accuracy.

To further highlight the damage maintained by T1DM and T2DMs, we investigated the mitochondrial structural profiles of these cell types while cultured in our 3D model. Mitochondrial dysfunction has been widely associated with altered cellular metabolism [41,42,45,70]. The loss of mitochondrial function and integrity has often been found to lead to various pathological conditions and diseases including diabetes, cardiovascular disorders, neurodegenerative disorders and different eye related diseases [71–73]. Unfortunately, not much information is available on the role of mitochondrial dysfunction with regards to diabetic keratopathy. Here, we report significant differences in mitochondrial structure in both T1DM and T2DMs, as compared to HCFs, when allowed to secrete and assembly their own ECM. These findings are in agreement with previous reports in the human corneal stroma showing that mitochondrial dysfunction [71] leads to collapsed inner mitochondrial membranes that may inhibit or activate the mitochondrial electron transport chain potential.

Corneal defects have been reported in both T1DM and T2DM patients, but the exact pathophysiological differences (if any) between the two is largely unknown. Future studies will aim to adress this and hope to pave the way for the devlopment of targeted therapeutics.

It is truly remarkable how both T1DM and T2DMs can maintain, at least partially, their inherent defects following *in vitro* expansion. Given the direct interplay of the corneal stroma and the nerve network we believe that key players to the regulation of the corneal DM defects exist in the stroma layer and the resident keratocytes/fibroblasts. Understanding the phenotype and characteristics of the human DM cornea stroma as well as the cells that reside within it is critical. We believe that our novel 3D model can dissect crucial aspects of the diabetic keratopathy pathology and pave the way for the development of new therapeutics.

## Conclusion

We strongly believe that our *in vitro* model will help our efforts to delineate the molecular mechanisms of the human diabetic keratopathy and pave the way for better understanding of the corneal stromal defects due to DM. Further development of this model should provide important clues to the development of novel therapeutics to treat diabetic keratopathy defects.
